# How to connect academics around the globe by organizing an asynchronous virtual unconference

**DOI:** 10.12688/wellcomeopenres.16893.2

**Published:** 2021-10-27

**Authors:** Constance Holman, Brianne A. Kent, Tracey L. Weissgerber

**Affiliations:** 1 QUEST Center, Berlin Institute of Health at Charité – Universitätsmedizin Berlin, Berlin, Berlin, 10117, Germany; 2 Department of Psychology, Simon Fraser University, Burnaby, British Columbia, V5A 1S6, Canada

**Keywords:** conference, unconference, virtual brainstorming, virtual platform

## Abstract

Many conferences and in-person meetings have transitioned to virtual platforms in response to the COVID-19 pandemic. Here, we share strategies and lessons learned from organizing an international virtual unconventional conference, or ‘unconference’. The event focused on how early career researchers can advocate for systemic improvements in scientific publishing and research culture. The virtual unconference had three main components: (1) a virtual networking event, (2) asynchronous virtual brainstorming, and (3) a virtual open space, where participants could join or lead in-depth discussions. The unconference format was participant-driven and encouraged dialogue and collaboration between 54 attendees from 20 countries on six continents. Virtual brainstorming allowed participants to contribute to discussions at times that were convenient for them. Activity was consistently high throughout the 48 hours of virtual brainstorming and continued into the next day. The results of these discussions are collaboratively summarized in a paper entitled *Empowering Early Career Researchers to Improve Science*, co-authored by the unconference participants *.* We hope that this method report will help others to organize asynchronous virtual unconferences, while also providing new strategies for participant-driven activities that could be integrated into conventional virtual conferences.

## Introduction

In response to the coronavirus disease 2019 (COVID-19) pandemic, academic conferences and workshops rapidly moved online. Virtual conferences reduce costs and lower an event’s carbon footprint, while providing equitable access to scientists who can’t travel due to limited funding, visa constraints, health issues, or family care obligations ^1^. The shift to virtual formats may have long-term benefits for early career researchers (ECRs) and scientists from countries with limited research funding, who face disproportionate obstacles in attending expensive, in-person events ^2^.

The rapid transition to virtual platforms due to the pandemic has made it even more important to share resources, best-practices, and new innovations ^2^. Unfortunately, conference organizers cannot simply re-create in-person programs online. Attendees often prefer shorter sessions, as many are expected to continue with their normal activities during the conference. Virtual conferences also need to accommodate participants who are spread across many time zones, by offering opportunities for asynchronous participation. In-person networking events are also difficult to execute online.

Scientists and conference organizers are currently testing new solutions to improve the virtual conference experience for participants. One example is Neuromatch ( neuromatch.io), a non-profit that has been working to improve conferences by utilizing matchmaking machine-learning algorithms to connect scientists with similar interests ^3, 4^. This approach allows researchers to expand their personal networks ^3^. In total, 3,000 attendees participated in the first virtual Neuromatch conference in March 2020, which included one-on-one meetings facilitated by a matchmaking algorithm. This helpful example illustrates how innovations can enhance the online conference experience for participants ^3, 4^.

The shift to online conferences builds upon other efforts to adapt scientific conferences, including the rise of participant-driven unconventional conferences, known as *un*conferences ^5^. Traditional conferences feature a rigid line-up of speakers presenting to the audience. In contrast, unconferences maximize the informal, stimulating discussions and networking that typically happen during coffee breaks at traditional conferences. Attendees collaboratively create content based on their shared interests and expertise. A science hackathon, where scientists work collaboratively on a new project for a short period of time, is an example of an unconference ^6^. 

Here we share our strategy for organizing an international virtual unconference that allowed for asynchronous participation. The unconference, which focused on how ECRs can advocate for systemic improvements in research culture and practice, included three components to facilitate collaborative, open, and inclusive discussions: (i) virtual networking events allowed participants to get to know each other, (ii) virtual asynchronous brainstorming ^7^ via an online discussion platform allowed participants to share ideas and experiences related to the conference themes, and (iii) a virtual open space allowed participants to organize or join in-depth breakout discussions, via videoconference, in real time. In this paper, we describe each of the three components of the virtual unconference in detail and discuss lessons learned.

## Methods

### Ethics statement

A formal ethics review was not required for this description of the unconference organization. Written informed consent of participants who shared their thoughts in the anonymous survey was obtained from the participants.

## Participants

Participants were selected based on their experience with ECR-driven initiatives to improve research culture and practice, with the goal of also having diverse representation. Participants were contacted personally via email by the event organizers several weeks before the event. This email contained information about the goals, time and format of the event, as well as a link to confirm participation and consent to share their ideas in a subsequent (extended data – VBE Survey 1) ^8^. Confirmed attendees were further asked to suggest other possible participants based on their experience in different sectors related to improving science and research culture (e.g., scientific publishing, social media advocacy, or initiatives in countries with limited research funding). Approximately one week prior to the event, confirmed participants were also encouraged to fill out an informal survey (extended data – VBE Survey 2) ^8^, outlining topics of interest, and optionally sharing their social media accounts. This information was posted prior to the event by the organizers to allow participants to identify others with similar interests or expertise.

*1. Virtual networking event*

Virtual networking events are essential to replace the in-person networking and discussions that are the centerpiece of an unconference. Our past experiences suggest that virtual brainstorming works best for groups where participants already know each other, so holding the virtual networking event prior to the brainstorming was a priority. We have found that some attendees may be uncomfortable sharing ideas and personal experiences online with acquaintances and strangers, even when these interactions occur on a private online forum where participants post under their own names and have agreed to adhere to the event’s code of conduct. Networking events, where participants get to know one another, may help participants to feel more comfortable engaging in the virtual brainstorming sessions. After meeting other attendees, participants are also able to tag those with interests and expertise that is relevant to a particular discussion.

We organized two virtual networking sessions to accommodate different schedules and time zones. The two times selected (6am EST and 12pm EST) targeted waking hours in time zones from Australia, China, continental Europe, Africa and the East coast of North and South America. Both events were held the day before the start of the 48-hour virtual brainstorming event. Each session began with a 15-minute webinar, where conference organizers welcomed participants and provided an overview of how to participate in a virtual brainstorming event. Organizers also explained how to use the virtual open space and gave instructions for the remainder of the networking event.

Once the introductory webinar was complete, participants moved to the virtual open space for a 45-minute networking session. One organizer stayed in the introductory video conference for 10 minutes to provide technical support for attendees who were having difficulty joining the open space. To create the open space, we used a free, online platform called Wonder ( Wonder.me). Briefly, on the Wonder platform participants enter a virtual room, where they appear as a small circular icon. Participants can move their icon anywhere in the virtual room. If their icon comes close to another icon or group of icons, the participant automatically enters a videoconference with others whose icons are close by. This flexible format allows many simultaneous small group discussions. Participants can move their icons to join a different conversation, break into smaller groups, or meet someone new. Participants were asked to limit group sizes to four people during the virtual networking event to ensure that everyone had an opportunity to talk. Other free platforms offer similar functionality (e.g., Gather ( Gather.town), SpatialChat ( spatial.chat)).

We encouraged participants to meet new people by changing the room background every seven to eight minutes (extended data – networking_images) ^8^. Each background asked participants to move about the room in a different way. Some backgrounds allowed participants to meet others with similar interests, whereas others facilitated “random” meetings. We used seven backgrounds during the event:

- **Opening background:** The entry background was a landscape photograph.
- **World map:** Participants were asked to go to the country where they live or work.
- **Random networking:** The third background asked participants to move around the room as quickly as possible for 15 seconds. After 15 seconds, the session organizer posted a new background asking participants to stop and talk to the person closest to them.
- **Themed content:** The fourth background asked participants what they would most like to change about science (i.e., open science, rewards and incentives, reproducibility, education, etc.). Participants were asked to move to the square listing the topic that they were most passionate about.
- **Maze (random networking):** Participants were asked to follow the maze until they met someone new.
- **Themed content:** The sixth background asked participants what obstacles they had faced as an ECR who wants to improve science. Attendees were asked to move to the square that best represented their answer (i.e., resistance to change, lack of time, lack of money or resources, power structures, and hierarchy, etc.).
- **Closing background:** At the end of the networking session, the organizer posted a background stating that the formal event had concluded, however, participants were welcome to stay for as long as they liked. The organizers stayed in the room to answer questions and many participants continued their conversations.

The different backgrounds were very effective in ensuring that participants had the opportunity to meet all others joining the session. The same virtual room was used as an open space for the rest of the event. Therefore, attendees were already comfortable using this space.

*2. Virtual brainstorming*

A Virtual Brainstorming Event is a group event where everyone discusses a particular topic or theme. The brainstorm is conducted on an online discussion platform (i.e., Discourse, Microsoft Teams, Slack, etc.) over a 24 to 48 hour period. Virtual Brainstorming Days are very effective for generating a lot of ideas quickly and identifying shared interests and expertise within a group. These events are designed to accommodate different schedules and time zones by enabling asynchronous participation. This was essential for our unconference, which included attendees on six continents and in many different time zones. Participants do not need to be online the whole time; instead, they can check in a few times per day to share their ideas and respond to others’ comments. Virtual brainstorming events are well-suited to topics that are too complex to resolve with a short discussion. This was also important for our unconference, which included five different topics related to a central theme.

Our virtual brainstorming event was organized following our previously described protocol ^7^, using Microsoft Teams. Prior to the event, participants received general instructions and tips for effective virtual brainstorming and a link to a five-minute video showing basic skills mentioned in the instructions. These included tips for navigating the online platform, replying to a post, reacting to a post, and tagging an individual or the entire group. Attendees were encouraged to log on a few days prior to the event to ensure that they had access to the discussion platform. The virtual brainstorming included five channels, each with a different discussion question. There were also three supporting channels: an introductions channel, a tech support channel, and a sign-up channel for the virtual open space. The five discussion questions were posted at the start of the event, and participants discussed these questions as well as other topics that arose over the next 48 hours. The event organizers monitored and participated in the discussion, following the tips for facilitators described in the protocol ^7^. After the first day of the event, organizers also met to review the posts to identify gaps in the discussion related to each of the five main themes. These gaps were addressed by posting questions or organizing open space sessions for the next day.

An additional advantage of virtual brainstorming is that it enables asynchronous participation, ensuring everyone has an opportunity to express their opinion, reflect on others’ posts and respond. This allows the conversation to evolve over the course of the event and provides time for participants to post links to or upload documents. Finally, virtual brainstorming levels the playing field by making it easier for everyone to contribute. This includes junior participants, participants who have limited expertise on the topic, and participants who are uncomfortable speaking up during live calls. Non-native speakers can use online translators to follow the discussion and have more time to prepare responses.

*3. Virtual open space*

The virtual brainstorming event was accompanied by a virtual open space to facilitate in-depth discussions. Participants could host sessions on any topic, at any time during the event. As in the networking session, we used Wonder ( wonder.me) for the virtual open space so that participants could break into smaller groups or rejoin the larger group at any time simply by moving their icons.

Participants were encouraged to use the open space for three different types of meetings: (i) discussions on topics that they were passionate about, (ii) coffee break style networking, or (iii) one-on-one conversations. Attendees announced open space events in a dedicated channel in the virtual brainstorm by posting the time of the session and topic of discussion. Some sessions addressed major themes of the unconference, whereas others developed out of conversations occurring in the virtual brainstorm. These sessions were also used to prepare resources.

Session organizers were asked to report back by posting information about what was discussed during the session. The informal summary template included four topics:

1. Who attended the discussion?
2. What key themes were discussed?
3. What were the main points of consensus and/or disagreement?
4. Did the conversation illuminate new perspectives, or serve as a jumping off point for new ideas and themes? If so, what new perspectives, ideas or themes were discussed?

Posting these informal summaries allowed participants who were unable to attend the session to add their thoughts after the session had concluded, sparking further discussion.

*4. Facilitating participation*

In this section, we describe several additional strategies that we used to engage participants.

One downside of vibrant virtual brainstorming events is that the number of posts and notifications can quickly become overwhelming. Participants who join a virtual brainstorming discussion later may have difficulty finding conversations relevant to their interests amidst the continuous flow of posts. To help participants identify where they can contribute, conference organizers met after the first day of brainstorming to tag participants who had not yet joined the discussion on posts relevant to their area of expertise. This also helped attendees to connect with other participants who had similar interests.

Some attendees did not have enough time to participate in the brainstorming due to unexpected competing obligations during the event. All participants were invited to continue replying to posts or sharing their opinions in the days following the brainstorming, with the understanding that these ideas would be included in the final manuscript summarizing the event. After the event, all participants were also sent an anonymous survey via email where they were asked to share their thoughts about the virtual brainstorming event (extended data - VBE_Survey3) ^8^. This was designed to help organizers identify strengths and weaknesses of the virtual brainstorming event, as well as aspects that could be improved-on for the future.

## Results

**Figure 1.**
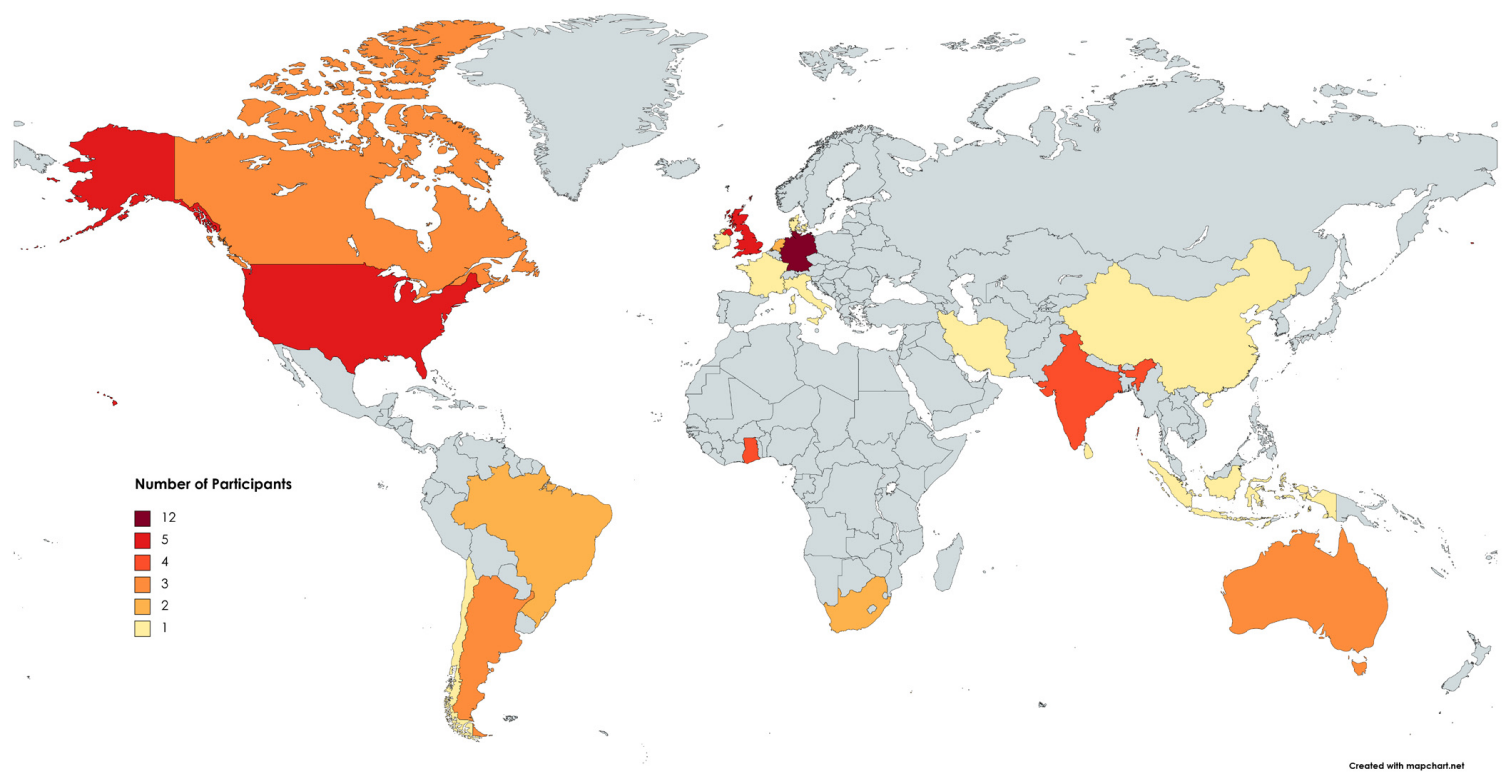
Scientists from 20 countries participated in the virtual unconference. Red countries represent the countries of participants’ home institutions at the time of the event. This figure was created using MapChart.net under a Creative Commons Attribution-ShareAlike 4.0 License ( https://mapchart.net/feedback.html).

Overall, 54 attendees from 20 countries on six continents ( Figure 1) participated in our virtual brainstorming event, which examined how ECRs can advocate for systemic improvements in research culture and practice. Activity was consistently high throughout the 48 hours of virtual brainstorming, and included 240 posts, 636 replies, 477 mentions, 507 reactions, and nine open space discussions. Some attendees continued to post and attend open space sessions in the days following the event. Two invitees who were interested in participating, but who reported being unavailable on the unconference dates in the initial survey (extended data – VBE_Survey1) ^8^, were offered the opportunity to answer the virtual brainstorming questions in advance. The conference organizers posted these comments on the discussion board on behalf of invitees during the first day of virtual brainstorming.

In addition to the main discussion, our organizers and participants also organized open space sessions. There were two open space sessions on the first day of the conference, five on the second day, and two additional sessions the day after virtual brainstorming had ended. The conference organizers moderated two sessions; the remainder were organized and run by conference participants. Open space sessions were organized anywhere from 30 minutes to 10 hours in advance, and typically lasted for about an hour. Informal summaries of these sessions were posted back on the main platform, where they were collaboratively viewed and edited by participants who were not able to attend the session.

In addition to discussion generated by these summaries, other outputs were also generated during open space sessions. For example, during one session run by an attendee, participants worked together to generate a list of digital tools that ECRs can use to make their research more reproducible. This list was posted on OSF (RRID:SCR_003238) ^9^ and shared on Twitter during the unconference.

After the event concluded, participants were also involved in preparing a manuscript summarizing the major themes discussed during the event ^10^. The five themes discussed during the conference corresponded to planned sections of the manuscript. This allowed organizers to identify areas where additional content was needed during the event and encourage further discussion. The event organizers shared an outline of the manuscript, followed by three drafts, with participants. All participants were invited to comment at each stage and the organizers worked to improve the outline and manuscript based on the feedback. In some cases, participants drafted specific sections on topics relevant to their expertise. Six event participants were not involved in editing the manuscript or chose not to appear on the authorship list.

Participants were also provided with an anonymous post-event survey to better understand their experience of the virtual brainstorming event, and to identify opportunities for improvement. 37/54 attendees shared their thoughts and provided suggestions for the future (VBE_Survey3_PostEventInfo_Responses) ^8^. Overall, nearly all (92%) of participants reported learning something new, planning to incorporate things that they learned into future work (75%), and meeting interesting potential collaborators (62%). The largest reported obstacles related to having limited time to participate (65%) and having scheduling conflicts which precluded participation in open space sessions (49%).

However, feedback also indicated that the open space sessions provided a valuable opportunity to explore topics raised in the virtual brainstorming discussions in depth. 97% of survey respondents found the software easy to use, and 75% indicated that they would likely use it again for their own work in the future. The organizers observed that these sessions also allowed participants to get to know one another in spontaneous, more organic ways. Several participants also communicated that open space sessions provided a useful alternative for attendees who preferred videoconferencing to text discussions.

The post-event survey also explored some limitations of the virtual brainstorming event format. Two of the most prominent obstacles mentioned by participants concerned time. Most participants reported planning two to four hours for participation in the event, but some reported devoting somewhat (22%) or much more time (11%) than they originally had planned. Our event ran for approximately 48 hours, however eight survey respondents commented that they would promote holding similar events over a longer period of time in the future. These considerations, including managing time costs related to information overload are discussed in more detail below.

## Discussion

We were pleased with the organization of the event, and thoroughly enjoyed reading posts and sharing ideas and experiences with participants. The results of our discussions are collaboratively summarized in a paper entitled *Empowering Early Career Researchers to Improve Science* ^10^. In the anonymous post event survey, almost all participants reported positive experiences. There were several important points which we feel contributed to the event’s success, and may be useful for others organizing similar events:

*Share the code of conduct with participants prior to the event:* This sets a clear expectation that the unconference should be a welcoming and inclusive space for participants to share ideas and experiences.

*Encourage participants to budget more time than they think that they will need:* While we asked participants to check in a few times per day, we observed that many participants were online more frequently. Keeping up with the intensive online discussions and open space events, in addition to normal workday activities, was challenging. Organizers may wish to emphasize that while checking in a few times per day is sufficient, many participants will want to do more. Scheduling time to participate fully will make the unconference more enjoyable.

*Address technical issues early by encouraging participants to log in prior to the event:* We encouraged participants to log on at least one day in advance to ensure that they could access the platform, view the information video, and explore the virtual brainstorming site. This allowed us to solve problems before the event.

*Get to know the conference participants before the event:* We used a pre-conference survey (extended data – VBE Survey 2) ^8^ to learn about participants’ activities surrounding the conference themes prior to the event. This made it easier to tag participants on posts that might be of interest to them.

*Technological information should be easy to find:* Many participants had no prior experience with one or both of the platforms that we used for this event. Five-minute videos were helpful in demonstrating basic skills. Links and passwords for each platform were clearly posted in the online discussion channel.

*Plan deliverables in advance and monitor progress during the event:* Some unconferences are designed to produce specific deliverables, whereas others aim to facilitate networking and discussion. Our goal was to write a paper on the conference theme, with all participants listed as authors. The five discussion themes each addressed a section of the planned paper. Organizers must balance the need to gather information for planned deliverables with the potentially competing desire to allow participants to explore topics that are interesting to them. Knowing one’s deliverables may also be important for planning and logistics. For example, if results from the event may be used for a peer-reviewed manuscript, it is important that participants understand and consent to this prior to participating. It is also important to have clear expectations about authorship and the participants’ role in preparing the manuscript.

*Help participants to run open space sessions during the first six hours:* Discussion and events in the open space were vital to the success of the event. Informal summaries from open space sessions generated many new ideas that strengthened discussions in the online brainstorming sessions. Conference organizers may wish to organize a few preplanned sessions and support a few attendees in organizing open space sessions within the first six hours of brainstorming. This builds momentum and gives others an example to follow when organizing their own sessions.

*Encourage participants to focus on discussions that align with their interests or expertise:* Virtual brainstorming events can quickly become overwhelming due to the large number of posts and replies. Let participants know that they don’t need to read every single post; they can focus their energy on topics where they can contribute the most.

*Ideas for improvements:* Unconference participants suggested several other strategies for improving on this format for future events. These include measures to combat “information overload” such as staggering discussion topics, extending the duration of the virtual brainstorming event, and asking moderators to post summaries at regular intervals throughout the event. Some participants requested more pre-planned open space sessions, as prior commitments made it difficult to join spontaneous open space sessions that were organized a few hours in advance. Balancing the amount or pre-planned and spontaneous content is always challenging for unconference organizers.

*Considerations for larger events*: The virtual brainstorming event included 54 participants, but we believe that this format may also be scaled-up to larger, more heterogeneous groups. There are several options in addition to the ideas for improvement, above, which may facilitate virtual brainstorming events for a larger crowd. First, events can include a mixture of discussion channels on general topics, where everyone is encouraged to contribute, and more specific channels geared toward special interest groups. The same approach may be taken with open space sessions, with a variety of scheduled sessions targeting all participants, or just a specific subset. Conference organizers should have multiple moderators available so that large groups in open space sessions can divide into smaller subgroups, allowing everyone to participate actively in the discussion. Finally, it is important to work with a team of moderators that meet at regular intervals. This can help distribute the work of monitoring discussion in multiple channels and posting periodic updates.

## Conclusions

While the transition from in-person conferences to virtual events has created challenges, it also offers an opportunity to develop innovative new formats that may benefit the global scientific community long after it is safe to resume travel. Expanding our ‘conference organization toolkit’ would allow the global scientific community to supplement traditional in-person or virtual conferences with more tailored events. The techniques used for an event would depend on the goals of the event, as well as the needs of its diverse participants. We hope that our strategies and lessons learned will help others to organize asynchronous virtual unconferences, while also providing new options for participant-driven activities that could be integrated into virtual conferences.

## Data availability statement

### Underlying data

 All data underlying the results are available in the OSF repository containing extended data and no additional source data are required.

### Extended data

Open Science Framework: How to connect academics around the globe by organizing an asynchronous virtual unconference. https://doi.org/10.17605/OSF.IO/R93PJ ^8^.

This project contains the following extended data:

- VBE_Survey1_Intake (a form to confirm participation in the event)
- VBE_Survey2_ParticipantInfo (a form to collect participant information and determine optimal times for networking events)
- VBE_Survey3_PostEventInfo (an anonymous form to collect participant impressions and data for optimizing design of future iterations)
- Networking_Images (a compressed folder containing background images from the networking event)
- VBE_Survey3_PostEventInfo_Responses (table of anonymized results from the post-event survey)
- Editable_VBE_Survey_Links (a docx file containing links to editable the above surveys)

Data are available under the terms of the Creative Commons Zero "No rights reserved" data waiver (CC0 1.0 Public domain dedication).
