# Supplementary material for: How to connect academics around the globe by organizing an asynchronous virtual unconference
Source: Wellcome Open Res. 2021 Oct 27;6:156. Originally published 2021 Jun 16. [Version 2] doi: 10.12688/wellcomeopenres.16893.2 (PMC8366301; doi:10.12688/wellcomeopenres.16893.2)
Supplement: Peer review history

# Peer review: Reviewer response for version 2

**DOI:** 10.21956/wellcomeopenres.19090.r46680

**Published:** 2022-02-25

**Authors:** Aziz Khan

**Affiliations:** 1 Stanford Cancer Institute, Stanford University, Stanford, CA, USA

The authors addressed all of my concerns/suggestions in the revised version.

Is the rationale for developing the new method (or application) clearly explained?

Yes

Is the description of the method technically sound?

Yes

Are the conclusions about the method and its performance adequately supported by the findings presented in the article?

Yes

If any results are presented, are all the source data underlying the results available to ensure full reproducibility?

No source data required

Are sufficient details provided to allow replication of the method development and its use by others?

Yes

Reviewer Expertise:

I am a computational biologist interested in gene regulation and regulatory genomics and epigenomics. I also advocate for open science, preprints, reproducibility in research and EDI in science.

I confirm that I have read this submission and believe that I have an appropriate level of expertise to confirm that it is of an acceptable scientific standard.

# Peer review: Reviewer response for version 1

**DOI:** 10.21956/wellcomeopenres.18635.r45335

**Published:** 2021-08-13

**Authors:** Elizabeth Kachur

**Affiliations:** 1 Medical Education Development, New York, NY, USA

This is a very well organized and informative paper that describes the authors’ experience with an “Unconference”. They share their methodology, evaluation results and insights about how to organize an asynchronous virtual conference. Actually, since the event also included some synchronous elements, a broader title would have been more accurate. Nonetheless, as we are heading towards blended or hybrid event formats in the post-COVID era, this paper is definitely a must-read for any conference organizer. It also provides a good number of resources such as links to innovative online programs that promote interaction among participants. The authors demonstrate a good understanding of how program designs can facilitate or hinder learning.

 The asynchronous format is especially advantageous for global collaborations since it is user-friendly for all participants, regardless of their time zone. The event model described should work best with a relatively small audience of experts who jointly work towards a deliverable, such as a position paper. The conference described included 54 attendees and resulted in a collaborative paper about “Empowering Early Career Researchers to Improve Science”. If the emphasis is on knowledge and skills acquisition, or if it is geared towards a much larger audience (e.g., with hundreds of attendees) this format may not be optimal. Nonetheless, elements of the described event can definitely be adapted to more traditional conferences whether they are virtual or in-person.  For example, it should be easy to integrate virtual networking by using one of the described programs (e.g., wonder.me) as a pre-conference activity. As the authors identified very accurately, socializing opportunities before a conference can enhance comfort and engagement during the conference.   

 This paper is easy to read and definitely provides some inspirations and practical guidance for moving away from “the traditional” to the “new normal” in conference organization.

Is the rationale for developing the new method (or application) clearly explained?

Yes

Is the description of the method technically sound?

Yes

Are the conclusions about the method and its performance adequately supported by the findings presented in the article?

Yes

If any results are presented, are all the source data underlying the results available to ensure full reproducibility?

Yes

Are sufficient details provided to allow replication of the method development and its use by others?

Yes

Reviewer Expertise:

I am a medical educator, working as a consultant with multiple institutions in the US and elsewhere.  I am also engaged in various professional organizations and have been involved in organizing virtual and in-person conferences for several decades.

I confirm that I have read this submission and believe that I have an appropriate level of expertise to confirm that it is of an acceptable scientific standard.

# Response

**Published:** 2021-10-01

**Authors:** Constance Holman

**Affiliations:** Charité-Universitätsmedizin Berlin, Germany

Dear Elizabeth, 

 Thank you very much for your positive review of this piece, and for sharing your insights and expertise in conference planning. We are glad that you found the piece interesting, and hope that it may prove useful for other readers. We would like to note that your point *“If the emphasis is on knowledge and skills acquisition, or if it is geared towards a much larger audience (e.g., with hundreds of attendees) this format may not be optimal”* was also shared by our other reviewer. While this is indeed new ground, we have added a few small points in the manuscript addressing the question of planning events for larger numbers of participants: 

*“* *Considerations for larger events: The virtual brainstorming event included 54 participants, but we believe that this format may also be scaled-up to larger, more heterogeneous groups. There are several options in addition to the ideas for improvement, above, which may facilitate virtual brainstorming events for a larger crowd. First, events can include a mixture of discussion channels on general topics, where everyone is encouraged to contribute, and more specific channels geared toward special interest groups. The same approach may be taken with open space sessions, with a variety of scheduled sessions targeting all participants, or just a specific subset. Conference organizers should have multiple moderators available so that large groups in open space sessions can divide into smaller subgroups, allowing everyone to participate actively in the discussion. Finally, it is important to work with a team of moderators that meet at regular intervals. This can help distribute the work of monitoring discussion in multiple channels and posting periodic updates.”* 

 Once again, thank you for taking time to review this piece and for sharing your thoughts with us. If you have any questions, comments, or concerns in the future, please do not hesitate to get in touch with us. 

 Sincerely, 

 The authors

# Peer review: Reviewer response for version 1

**DOI:** 10.21956/wellcomeopenres.18635.r44510

**Published:** 2021-07-06

**Authors:** Aziz Khan

**Affiliations:** 1 Stanford Cancer Institute, Stanford University, Stanford, CA, USA

The unconventional conferences, aka “ *unconferences*” are now known for decades, and unlike conventional conferences, these provide participants the freedom to create an event they wanted.

 Most of the traditional conferences went virtual due to the ongoing COVID-19 pandemic and hopefully, these will partially or fully stay virtual even after the pandemic. Given virtual conferences and unconferences have many benefits, it is essential to showcase such virtual events’ success stories and provide tools, templates, strategies, and guidelines for other communities to adapt.

 In this paper, authors shared their experiences after organizing a participant-driven virtual unconference of 54 early career researchers (ECRs) from 20 countries. The organizers divided the event into three main parts: (i) networking (ii) brainstorming (ii) open discussions. These three components are further described with tools and suggestions for other communities organizing similar events. The outputs from this unconference event are presented in yet another important paper calling for action to improve science by empowering the ECRs (https://doi.org/10.31219/osf.io/p5evw). ^1^ I believe that the experiences shared in this article are useful and will help other communities to organize asynchronous virtual unconferences and potentially integrate into the emerging virtual conferences.

 The manuscript is detailed, organized, and well-written. I have some minor comments:

1. Given virtual brainstorming events can be overwhelming – how to tackle such problems while scaling up the participation? The unconference described in this article is ECR specific with a relatively small number of participants (n=54). Can authors provide recommendations to scale up such virtual events with larger groups of scientists of different career levels and locations while making it less overwhelming?
2. In Figure 1, it will be good to use a color scale to show the number of participants from each of the 20 countries.
3. The survey forms are shared as PDF copies. If possible, please share copyable links for the Google forms so the community can reuse those with minimal modifications to host related events.
4. In the first sentence of introduction section, the correct abbreviation of COVID-19 is Coronavirus disease 2019.

Is the rationale for developing the new method (or application) clearly explained?

Yes

Is the description of the method technically sound?

Yes

Are the conclusions about the method and its performance adequately supported by the findings presented in the article?

Yes

If any results are presented, are all the source data underlying the results available to ensure full reproducibility?

No source data required

Are sufficient details provided to allow replication of the method development and its use by others?

Yes

Reviewer Expertise:

I am a computational biologist interested in gene regulation and regulatory genomics and epigenomics. I also advocate for open science, preprints, reproducibility in research and EDI in science.

I confirm that I have read this submission and believe that I have an appropriate level of expertise to confirm that it is of an acceptable scientific standard.

## References

[1] : Empowering Early Career Researchers to Improve Science.2021; 10.31219/osf.io/p5evw 10.31219/osf.io/p5evw

# Response

**Published:** 2021-10-01

**Authors:** Constance Holman

**Affiliations:** Charité-Universitätsmedizin Berlin, Germany

Dear Aziz, 

 Thank you very much for your kind comments and helpful suggestions related to this manuscript. We agree with all of the minor points that you have raised, and have made the following changes: 

**Given virtual brainstorming events can be overwhelming – how to tackle such problems while scaling up the participation? The unconference described in this article is ECR specific with a relatively small number of participants (n=54). Can authors provide recommendations to scale up such virtual events with larger groups of scientists of different career levels and locations while making it less overwhelming?**

 Thank you for the suggestion. We have added the following paragraph providing suggestions for larger events in the discussion section:

*“* *Considerations for larger events: The virtual brainstorming event included 54 participants, but we believe that this format may also be scaled-up to larger, more heterogeneous groups. There are several options in addition to the ideas for improvement, above, which may facilitate virtual brainstorming events for a larger crowd. First, events can include a mixture of discussion channels on general topics, where everyone is encouraged to contribute, and more specific channels geared toward special interest groups. The same approach may be taken with open space sessions, with a variety of scheduled sessions targeting all participants, or just a specific subset. Conference organizers should have multiple moderators available so that large groups in open space sessions can divide into smaller subgroups, allowing everyone to participate actively in the discussion. Finally, it is important to work with a team of moderators that meet at regular intervals. This can help distribute the work of monitoring discussion in multiple channels and posting periodic updates.”* 

**In Figure 1, it will be good to use a color scale to show the number of participants from each of the 20 countries.**

 Thank you for this suggestion. We have updated the map with an approximate colour scale representing the number of participants for each country.

**The survey forms are shared as PDF copies. If possible, please share copyable links for the Google forms so the community can reuse those with minimal modifications to host related events.**

 We have added a new document to our online repository ( https://osf.io/qtpz7/ ), which included links to editable versions of the surveys included in this piece.**

**In the first sentence of introduction section, the correct abbreviation of COVID-19 is Coronavirus disease 2019.**

 Fixed – thank you! 

 Once again, we are very grateful for the points you have raised in your review, and believe that they have improved the manuscript and supplementary materials. Should you have any further comments or concerns, please do not hesitate to get in contact. 

 Sincerely, 

 The authors
